# Early radiographic detection and clinical implications of third-molar agenesis in Turkish children and adolescents

**DOI:** 10.4317/medoral.28129

**Published:** 2026-03-07

**Authors:** Melda Pelin Akkitap, Eser Rengin Nalbantoglu

**Affiliations:** 1 Department of Oral and Maxillofacial Radiology, Faculty of Dentistry, Biruni University, Istanbul, Turkey; 2 Department of Paediatric Dentistry, Faculty of Dentistry, Biruni University, Istanbul, Turkey

## Abstract

**Background:**

Third-molar agenesis represents one of the most common developmental dental anomalies and has important clinical implications for orthodontic treatment planning and space management. Numerous epidemiological studies have evaluated third-molar agenesis worldwide; however, contemporary data based on standardized radiographic criteria in Turkish paediatric populations remain limited. This study aimed to evaluate the prevalence and early detection patterns of third-molar germ agenesis in Turkish children and adolescents.

**Material and Methods:**

Panoramic radiographs of 1,570 individuals aged 9-18 years (811 females, 759 males) were retrospectively evaluated. Agenesis was diagnosed when no radiolucency corresponding to the osseous crypt was observed and no extraction history was present. All images were assessed by two calibrated examiners demonstrating excellent inter-examiner agreement (=0.90). Prevalence and distribution patterns were analysed according to jaw, side, gender, and age group (9-12 vs. 13-18 years) using Chi-square and Mann-Whitney U tests (p&lt;0.05). Multivariate logistic regression analysis was performed to identify independent predictors of third-molar agenesis, and adjusted odds ratios with 95% confidence intervals were calculated.

**Results:**

Of 6,280 third-molar sites evaluated, 1,312 (20.9%) demonstrated agenesis. At the individual level, 33.1% of participants had at least one missing third-molar. Agenesis was more frequent in the maxilla (24.9%) than in the mandible (16.9%). The most common pattern was bilateral absence of two molars (11.7%). Agenesis prevalence was significantly higher in the 9-12-year group across all tooth regions (p&lt;0.001).

**Conclusions:**

Third-molar germ agenesis was common in Turkish children and adolescents. Age- and jaw-specific distribution patterns support early radiographic assessment during late mixed dentition and may provide clinically relevant information for orthodontic treatment planning while supporting ALARA(As Low As Reasonably Achievable)-based imaging strategies.

## Introduction

Phenotypic plasticity allows humans to adapt to environmental, dietary, and developmental pressures, thereby influencing craniofacial morphology throughout evolution (1 , 2). Transitions toward softer diets and reduced masticatory demands have been associated with decreased jaw size and increased variability in third-molar development, including impaction and agenesis (3). As the last teeth to form, third-molars are particularly susceptible to genetic and environmental influences and demonstrate the highest frequency of agenesis among permanent teeth (4). These developmental changes are not only of evolutionary interest but also have important clinical implications for orthodontic diagnosis and long-term treatment planning.

Tooth agenesis, defined as the congenital absence of one or more permanent teeth, represents one of the most common developmental anomalies with multifactorial etiology (5 , 6). Third-molar agenesis may occur up to 13 times more frequently than agenesis affecting other teeth, with global prevalence estimates ranging from 9% to 41%, depending on population characteristics, genetic background, and diagnostic criteria (4 , 7). Recent evidence suggests a possible increasing prevalence trend, which may reflect ongoing evolutionary adaptation (6). Furthermore, emerging genomic studies have identified candidate loci associated with odontogenesis and craniofacial morphology, highlighting the biological and developmental significance of third-molar agenesis (8 , 9).

Although numerous international investigations have documented patterns of third-molar agenesis, evidence from Turkey-particularly in paediatric and adolescent populations-remains heterogeneous. Existing Turkish studies vary considerably in terms of age ranges, sample size, imaging protocols, diagnostic thresholds, and examiner calibration, which limits comparability and generalizability of findings (10 - 13). Moreover, relatively few studies have specifically evaluated early detection patterns during late mixed dentition, a developmental stage in which third-molar crypt formation becomes radiographically detectable and may influence orthodontic treatment timing and space management decisions (7 , 14 , 15). Recent studies have emphasized that panoramic assessment during late mixed dentition can help predict third-molar development (11). Nevertheless, delayed odontogenesis may mimic agenesis in younger individuals and should be considered in interpretation (16).

Therefore, the present study aimed to determine the prevalence and distribution of third-molar germ agenesis in Turkish children and adolescents using standardized panoramic radiographic criteria and calibrated examiner assessment. By analysing age-, gender-, jaw-, and side-specific patterns, this study seeks to provide contemporary population-based data with potential clinical relevance for early orthodontic planning and public health considerations, while contributing to strategies aimed at reducing unnecessary radiographic exposure in accordance with ALARA principles.

## Material and Methods

This retrospective radiological study was approved by the Research Ethics Committee of Biruni University Faculty of Dentistry (protocol no. 2025-BAEK/13-37). The study was conducted at the Radiology Unit of Biruni University Faculty of Dentistry between January 2024 and June 2025. A total of 1,570 patients who attended the clinic for various dental reasons and required panoramic radiography for diagnostic and treatment planning purposes were included. No additional radiographic exposures were obtained for research purposes, and all procedures were performed in accordance with ALARA principles. Written informed consent was obtained from all participants or their legal guardians.

The study population consisted of systemically healthy individuals aged 9 to 18 years who had available panoramic radiographs and no history of surgical intervention in the posterior regions of either jaw. Radiographs were excluded if they contained artifacts, were of insufficient diagnostic quality, or belonged to individuals with a history of orthodontic treatment, third-molar extraction, or germectomy.

An a priori power analysis was performed using G*Power (version 3.1, Heinrich-Heine-Universität Düsseldorf, Germany) to estimate the minimum required sample size. Sample size calculation assumed 95% statistical power, a 5% significance level, and an effect size of 0.3, yielding a minimum requirement of 347 subjects. The final study population consisted of 1,570 individuals, exceeding the required sample size and ensuring adequate statistical power.

**Figure 1:**
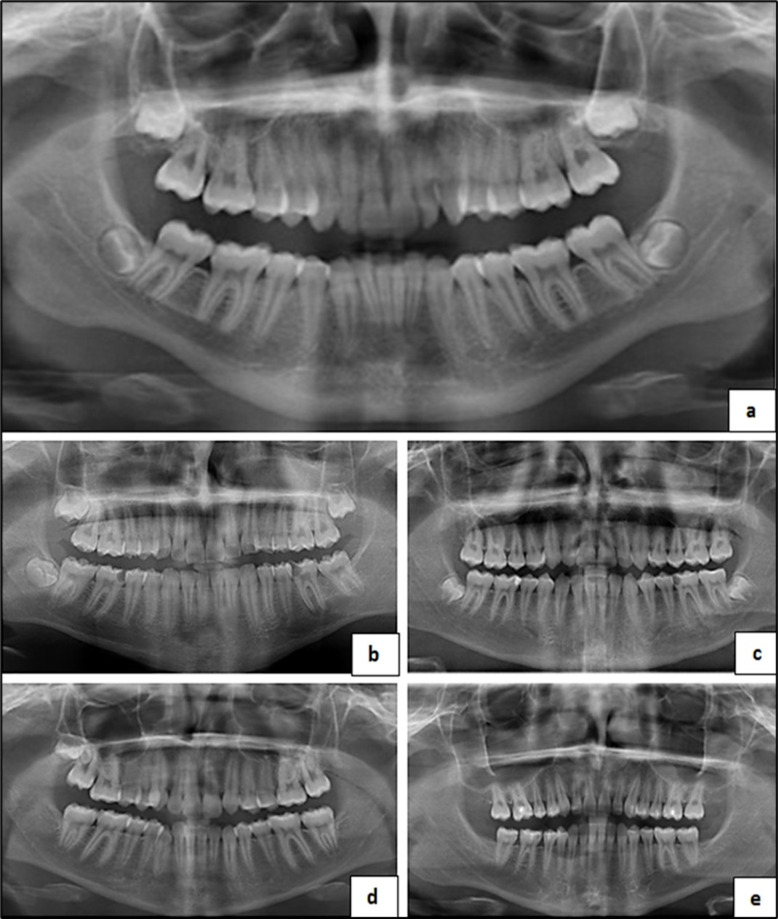
Panoramic radiographs showing third-molar agenesis patterns: (a) all third-molar germs present, (b) single agenesis, (c) double agenesis, (d) triple agenesis and (e) total agenesis.

**Figure 2:**
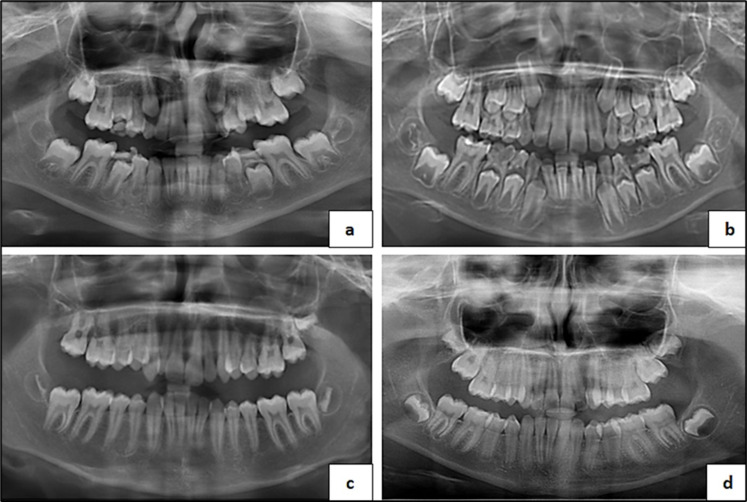
Panoramic radiographs showing third-molar germs at different ages: (a) 9 years, (b) 10 years, (c) 11 years and (d) 12 years.

Third-molar agenesis was defined based on age-related expectations of dental germ development. Crypt formation of third-molars generally begins between 7-9 years in the maxilla and 8-10 years in the mandible, with eruption typically occurring between 17-21 years and root completion by 18-25 years (14). In the present study, a third-molar was considered congenitally absent when no radiolucency corresponding to the osseous crypt was observed on panoramic radiographs and no evidence of extraction was present (15) (Figure 1). Individuals aged 9-18 years were included, as third-molar crypt formation is typically radiographically detectable by the age of nine and eruption generally begins around 18 years (Figure 2). For chronological analysis, participants were categorized into two groups: 9-12 years (mixed dentition) and 13-18 years (permanent dentition). The prevalence of third-molar agenesis was evaluated according to age, gender, symmetry, and jaw involvement.

1

2

Panoramic radiographs were obtained using an orthopantomography device (Sirona ORTHOPHOS XG, Sirona, Bensheim, Germany) operating at 60-80 kVp, 8-10 mA, and 12.8-second exposure time, with a magnification factor of 1.2. Images were evaluated on a calibrated monitor under low ambient lighting conditions with standardized brightness and contrast adjustments. If agenesis could not be reliably determined, the radiograph was excluded from analysis. All images were independently evaluated by an experienced oral and maxillofacial radiologist and a paediatric dentist. To assess inter-examiner reliability, 10% of the radiographs were randomly re-evaluated after a two-week interval. Inter-examiner agreement was excellent (=0.90).

Statistical analyses were performed using SPSS software (version 27.0; IBM Corp., Armonk, NY, USA). Data distribution normality was assessed using histogram plots and the Kolmogorov-Smirnov test. Descriptive statistics were presented as mean, standard deviation, median, and minimum-maximum values. Categorical variables were compared using the Chi-square test, whereas comparisons between two groups for non-normally distributed variables were performed using the Mann-Whitney U test. A p-value of less than 0.05 was considered statistically significant (p&lt;0.05). To identify independent predictors of third-molar agenesis, multivariate logistic regression analysis was performed. Age group (9-12 vs. 13-18 years) and gender (female vs. male) were included as independent variables. Adjusted odds ratios (ORs) with 95% confidence intervals (CIs) were calculated.

## Results

**Figure 3:**
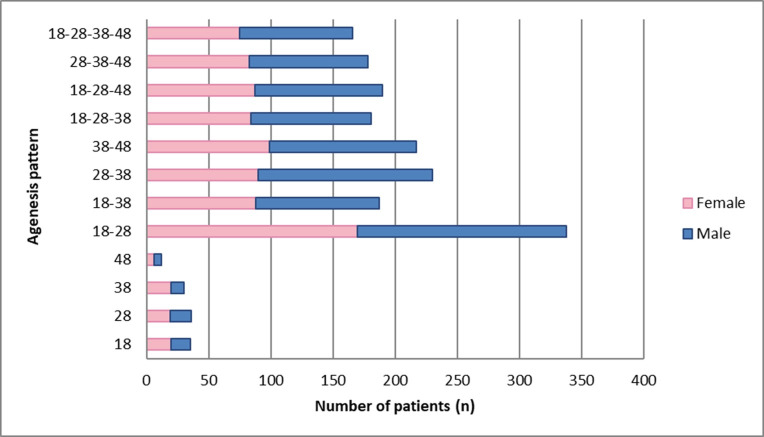
Distribution of third-molar agenesis patterns according to gender. The figure illustrates the frequency of agenesis combinations involving teeth 18, 28, 38 and 48 according to the FDI numbering system, highlighting symmetry patterns and gender-related distribution differences across quadrants.

In total, 6,280 third-molar sites from 1,570 panoramic radiographs (811 females, 759 males) were analysed. The participants' ages ranged from 9 to 18 years (mean: 12.92±2.96 years). Of the total sample, 766 individuals (48.79%) were in the 9-12-year age group and 804 (51.21%) were in the 13-18-year age group. Of these sites, 1,312 (20.9%) demonstrated agenesis. At the individual level, 519 of 1,570 participants (33.1%) exhibited agenesis of at least one third-molar. The most frequent pattern was agenesis of two third-molars, observed in 183 individuals (11.7%), followed by agenesis of four third-molars (166, 10.6%), agenesis of one third-molar (114, 7.3%), and agenesis of three third-molars (56, 3.6%). Of these individuals, 254 were male and 265 were female, indicating a slightly higher prevalence among females (Figure 3).

3

Regarding jaw involvement, third-molar agenesis was more common in the maxilla, 781 (24.9%), than in the mandible, 531 (16.9%). However, no significant gender differences were observed (p=0.539 and p=0.528, respectively; p&gt;0.05) (Table 1).

Table 1In terms of laterality, the prevalence of agenesis was similar between the right (20.7%) and left (21.1%) sides, with no statistically significant side predilection (p=0.710 and p=0.893, respectively; p&gt;0.05). Likewise, no significant gender differences were found for either side (p=0.115 and p=0.256, respectively; p&gt;0.05) (Table 2).

Table 2According to the tooth number, the most common third-molar agenesis was detected in the left maxillary third-molar (28; 25.16%), followed by the right maxillary third-molar (18; 24.59%), left mandibular third-molar (38) (17.01%), and right mandibular third-molar (48; 16.83%). When third-molar agenesis was compared by gender at the tooth number, slightly higher frequencies were observed in females for the left maxillary (28) and left mandibular (38) teeth, and in males for the right maxillary (18) and right mandibular (48) teeth. However, this difference reached statistical significance only for the right mandibular third-molar (48) (p=0.037; p&lt;0.05) (Table 3). At the tooth level, the proportion of agenesis was significantly lower in the 13-18-year group than in the 9-12-year group for all tooth regions (p&lt;0.001) (Table 4).

Table 3

Table 4Multivariate logistic regression analysis revealed that age group was a significant independent predictor of third-molar agenesis. Individuals aged 9-12 years had significantly higher odds of agenesis compared with those aged 13-18 years (OR= 2.32, 95% CI= 1.86-2.88, p&lt;0.001). No significant association was observed between gender and third-molar agenesis (OR= 0.99, 95% CI= 0.79-1.23, p=0.902).

## Discussion

This large-scale radiographic study provides updated epidemiological evidence regarding third-molar germ agenesis in Turkish children and adolescents. The individual-level prevalence observed in the present cohort (33.1%) falls within the upper range of global prevalence values reported in recent systematic and population-based investigations (4 , 7 , 17). The prevalence observed in the present study is comparable to findings from several contemporary radiographic epidemiological studies, while being slightly higher than those reported in a recent study conducted in Turkey (10). Such variations may reflect regional genetic diversity, environmental influences, dietary transitions, and methodological differences, including variations in diagnostic thresholds, age cohorts, and imaging protocols.

Age-related differences represented one of the most important findings in this study. The significantly higher prevalence observed in the 9-12-year age group supports previous research suggesting that third-molar agenesis may be identifiable shortly after the expected crypt formation period (14 , 15). Lower prevalence in older individuals may reflect improved visualization of developing tooth germs and reduced diagnostic uncertainty. These findings emphasize the potential clinical relevance of evaluating third-molar development during late mixed dentition, when orthodontic treatment planning and long-term space management decisions are frequently considered.

Jaw-related findings in the present study demonstrated a higher prevalence of agenesis in the maxilla compared with the mandible. This distribution pattern is consistent with the majority of epidemiological investigations conducted across European, Asian, and Mediterranean populations (11 , 18). These findings have been associated with evolutionary reductions in maxillary arch length, altered craniofacial growth dynamics, and differential susceptibility of maxillary third-molars to environmental and genetic influences (9).

The absence of consistent gender differences across most tooth regions observed in the present study is consistent with several previous investigations (7 , 11 , 13). Although isolated gender-related variations have been reported in certain populations (18 - 20), such findings remain inconsistent and are likely influenced by sample size variability, population ancestry, and methodological inclusion criteria. In the present cohort, the statistically significant difference observed only for tooth #48 may represent localized developmental variation rather than a generalized biological pattern.

Analysis of agenesis distribution patterns according to the number of missing teeth demonstrated that bilateral agenesis patterns were the most common. Similar symmetrical distribution patterns have been reported in several international studies and may reflect genetic regulation of dental development and odontogenesis symmetry (18 , 21). Bilateral agenesis is generally considered to suggest a stronger hereditary component compared with unilateral absence patterns.

Recent epidemiological investigations have confirmed considerable population variability in third-molar agenesis prevalence (15 , 22). Previous radiographic investigations in Turkish populations have emphasized the clinical importance of panoramic imaging in identifying developmental dental anomalies and supporting orthodontic evaluation (12).

Radiographic reliability studies have further highlighted the importance of early panoramic assessment during late mixed dentition. Dumas et al. (15) demonstrated that the absence of third-molar crypt formation during childhood may provide clinically meaningful predictive information for orthodontic treatment planning. However, early diagnosis should be interpreted cautiously, as delayed odontogenesis may mimic agenesis patterns in younger individuals, as reported by León-Rubio et al. (16).

Recent advances in genetic research have also contributed to understanding the biological mechanisms underlying dental agenesis. Molecular investigations conducted by Modafferi et al. (8) identified several candidate pathways influencing odontogenesis and craniofacial morphology, supporting the multifactorial nature of third-molar agenesis, which may also coexist with other developmental dental anomalies (23).

The present study contributes updated epidemiological data on third-molar agenesis based on a large paediatric cohort evaluated using standardized panoramic radiographic criteria and calibrated examiner assessment. Inclusion of younger age groups enabled evaluation of early radiographic detection patterns that remain relatively underrepresented in current literature. Early assessment of third-molar development may provide clinically relevant information for orthodontic treatment planning. While visualization of third-molar crypt formation generally supports routine developmental monitoring, absence of crypt formation during late mixed dentition may indicate a higher likelihood of agenesis; however, delayed odontogenesis should be considered, particularly in younger individuals. Therefore, clinical decision-making should rely on individualized evaluation of radiographic findings, developmental stage, and orthodontic requirements, while minimizing unnecessary radiographic exposure in accordance with ALARA principles and supporting evidence-based orthodontic treatment planning.

Nevertheless, several limitations should be acknowledged. The retrospective cross-sectional design limits differentiation between true agenesis and delayed odontogenesis, particularly in younger individuals. Radiographic identification of third-molar crypt formation may also be influenced by individual variation in mineralization timing, which may introduce potential misclassification bias. Additionally, the single-center urban sample may limit generalizability to the broader Turkish population. Future multicenter longitudinal studies incorporating genetic, environmental, and socioeconomic variables are warranted to further clarify developmental variability and improve predictive diagnostic accuracy.

## Conclusions

Third-molar germ agenesis was common in this large cohort of Turkish children and adolescents, with prevalence values within the upper range of global reports. The findings provide updated epidemiological evidence based on standardized radiographic assessment in a paediatric population. Age- and jaw-specific distribution patterns suggest that third-molar agenesis may be identifiable from approximately 9 years of age. Absence of third-molar crypts on clinically indicated panoramic radiographs during late mixed dentition may provide valuable information for space management, anchorage planning, and treatment decision-making. Early recognition may also support ALARA-based strategies by potentially reducing unnecessary radiographic follow-up and surgical interventions.

## Figures and Tables

**Table 1:** Table Distribution of third-molar agenesis according to jaw and gender.

Jaw	Gender	Third-molar agenesis				p-value
		Absent		Present		
		n	%	n	%	
Maxilla	Female	1226	51.97	396	50.70	0.539
	Male	1133	48.03	385	49.30	
Mandible	Female	1341	51.42	281	52.92	0.528
	Male	1267	48.58	250	47.08	

Pearson's chi-square test. Significance at p<0.05. n: Number of teeth. %: Percentage.

**Table 2:** Table Distribution of third-molar agenesis by side and gender.

Side	Gender	Third-molar agenesis				p-value
		Absent		Present		
		n	%	n	%	
Right	Female	1304	52.39	318	48.92	0.115
	Male	1185	47.61	332	51.08	
Left	Female	1293	52.18	329	49.70	0.256
	Male	1185	47.82	333	50.30	

Pearson's chi-square test. Significance at p<0.05. n: Number of teeth. %: Percentage.

**Table 3:** Table Distribution of third-molar agenesis by gender and tooth number.

Tooth	Gender	Third-molar agenesis				p-value
		Absent		Present		
		n	%	n	%	
#18	Female	614	75.71	197	24.29	0.779
	Male	570	75.10	189	24.90	
#28	Female	612	75.46	199	24.54	0.557
	Male	563	74.18	196	25.82	
#38	Female	681	83.97	130	16.03	0.287
	Male	622	81.95	137	18.05	
#48	Female	690	85.08	121	14.92	0.037
	Male	616	81.13	143	18.87	

Pearson's chi-square test. Significance at p<0.05. n: Number of teeth. %: Percentage. According to the FDI system, 18, 28, 38 and 48 denote the right maxillary, left maxillary, left mandibular and right mandibular third-molars, respectively.

**Table 4:** Table Distribution of third-molar agenesis by tooth number and age group.

Tooth	Age (years)	Third-molar agenesis				p-value
		Absent		Present		
		n	%	n	%	
#18	9-12	493	64.36	273	35.64	<0.001
	13-18	691	85.95	113	14.05	
#28	9-12	480	62.66	286	37.34	<0.001
	13-18	695	86.44	109	13.56	
#38	9-12	603	78.72	163	21.28	<0.001
	13-18	700	87.06	104	12.94	
#48	9-12	594	77.52	172	22.48	<0.001
	13-18	712	88.56	92	11.44	

Pearson's chi-square test. Significance at p<0.05. n: Number of teeth. %: Percentage. According to the FDI system, 18, 28, 38 and 48 denote the right maxillary, left maxillary, left mandibular and right mandibular third-molars, respectively. Age-group comparisons were performed at the tooth level.

## Data Availability

Declared none.
